# Increased Risk of Hepatocellular Carcinoma and Liver Cirrhosis in Vinyl Chloride Workers: Synergistic Effect of Occupational Exposure with Alcohol Intake

**DOI:** 10.1289/ehp.6972

**Published:** 2004-05-27

**Authors:** Giuseppe Mastrangelo, Ugo Fedeli, Emanuela Fadda, Flavio Valentini, Roberto Agnesi, Giancarlo Magarotto, Teresio Marchì, Andrea Buda, Massimo Pinzani, Diego Martines

**Affiliations:** ^1^Department of Environmental Medicine and Public Health, University of Padua, Padova, Italy; ^2^Occupational Health Service, Local Health Unit 13, Dolo, Italy; ^3^Occupational Health Service, Local Health Unit 12, Venice, Italy; ^4^Department of Surgical and Gastroenterological Sciences, University of Padua, Italy; ^5^Department of Internal Medicine, University of Florence, Italy

**Keywords:** alcohol, case–referent studies, cirrhosis, hepatocellular carcinoma, occupational diseases, vinyl chloride

## Abstract

Hepatocellular carcinoma (HCC) and liver cirrhosis (LC) are not well-established vinyl chloride monomer (VCM)–induced diseases. Our aim was to appraise the role of VCM, alcohol intake, and viral hepatitis infection, and their interactions, in the etiology of HCC and LC. Thirteen cases of HCC and 40 cases of LC were separately compared with 139 referents without chronic liver diseases or cancer in a case–referent study nested in a cohort of 1,658 VCM workers. The odds ratios (ORs) and the 95% confidence intervals (CIs) were estimated by common methods and by fitting models of logistic regression. We used Rothman’s synergy index (*S*) to evaluate interactions. By holding the confounding factors constant at logistic regression analysis, each extra increase of 1,000 ppm × years of VCM cumulative exposure was found to increase the risk of HCC by 71% (OR = 1.71; 95% CI, 1.28–2.44) and the risk of LC by 37% (OR = 1.37; 95% CI, 1.13–1.69). The joint effect of VCM exposure above 2,500 ppm × years and alcohol intake above 60 g/day resulted in ORs of 409 (95% CI, 19.6–8,553) for HCC and 752 (95% CI, 55.3–10,248) for LC; both *S* indexes suggested a synergistic effect. The joint effect of VCM exposure above 2,500 ppm × years and viral hepatitis infection was 210 (95% CI, 7.13–6,203) for HCC and 80.5 (95% CI, 3.67–1,763) for LC; both *S* indexes suggested an additive effect. In conclusion, according to our findings, VCM exposure appears to be an independent risk factor for HCC and LC interacting synergistically with alcohol consumption and additively with viral hepatitis infection.

Although a large body of evidence from experimental and epidemiologic studies has demonstrated the relationship between exposure to vinyl chloride monomer (VCM) and angiosarcoma [International Agency for Research on Cancer (IARC) 1987; Lee et al. 1996], there is little evidence of a causal association between VCM and hepatocellular carcinoma (HCC) and liver cirrhosis (LC).

In their study on the U.S. cohort of VCM-exposed workers, Mundt et al. (2000) found an increased risk of liver cancer, mainly liver angiosarcomas. In the study, however, they distinguished HCC from angiosarcoma on the basis of information on the cause of death reported in death certificates. In the European cohort of VCM workers, Ward et al. (2001) searched for the best evidence of liver cancer by reviewing all available documentation and found a marked exposure–response relationship for all liver cancers (71 cases), angiosarcoma (37 cases), and HCC (10 cases). This evidence is also inconclusive because the number of HCC cases was small, there was a disproportionate excess of liver cancers with “other and unknown histology,” and the risk estimates were not adjusted for the influence of well-known risk factors for HCC: alcohol consumption and viral infection. Recently Wong et al. (2003) suggested an interaction between occupational VCM exposure and hepatitis B virus (HBV) infection in the development of liver cancer.

Data on an association between VCM and LC are even scarcer and are inconclusive. Du and Wang (1998) reported a significantly increased number of hospital admissions among Taiwanese VCM workers due to primary liver cancer and cirrhosis of the liver. In the European cohort of vinyl chloride workers, Ward et al. (2001) reported that overall mortality from cirrhosis was decreased, although there was a trend toward an increase in cirrhosis mortality proportionate to an increase in cumulative exposure. In this case, risk estimates were not adjusted for the confounding influence of alcohol consumption and HBV infection.

Pirastu et al. (2003) reported on a cohort of 1,658 workers employed in a VCM manufacturing plant, in which the standardized mortality ratio (SMR) for primary liver cancer of 2.78 was significantly increased. Because cohort studies are unavoidably affected by selection (healthy worker effect), information (misclassification of exposure and diagnosis of diseases based on death certificate), and confounding biases [alcohol intake, HBV/hepatitis C virus (HCV) carrier status], we carried out a case–referent study nested in the same cohort. In northeast Italy (Porto Marghera, Venice), where the plant is located, alcohol consumption is heavy and viral hepatitis common. These particular exposure conditions appeared suitable for the appraisal of the individual role of VCM exposure, alcohol intake, viral hepatitis infections, and their interactions in the etiology of HCC and LC.

## Materials and Methods

The present case–referent study was carried out on the occasion of a lawsuit by hundreds of workers, local municipalities, and the Italian national government against the VCM plant management. At the beginning of the lawsuit, the company indemnified any health problem that claimant workers themselves attributed to their past exposure in the plant. Among the “claimants” were 13 cases of HCC [8 confirmed by histology and 5 based on the criteria recently issued by the Italian Association for the Study of the Liver and the British Society of Gastroenterology (Ryder 2003)—focal hepatic lesions at sonography and α1-fetoprotein > 400 μg/L (Ryder 2003)], and 40 cases of LC (24 with histologic confirmation and 16 with clinical evidence of portal hypertension, ascites, and/or esophageal varices). Out of the 13 HCC cases, 11 also had LC and are included in the series of LC cases.

We found information on diagnosis in hospital records, which we actively searched for deceased subjects (vital status and cause of death were ascertained for all the cohort members through 1999); incident cancer cases (ascertained through the regional cancer registry for all the cohort members from 1987 to 1999); and all other claimant workers.

Six hundred and forty-three former VCM workers belonging to the above cohort were examined from 1999 through 2002 by occupational physicians at the Occupational Health Services (OHS) of two local health authorities in the course of a medical surveillance program launched by the Regione Veneto and the Italian Ministry of Health. Among these subjects, we identified 139 subjects without clinical (including liver sonography) or biochemical (normal serum levels of aspartate aminotransferase, alanine aminotransferase, and γ-glutamyl transpeptidase) evidence of chronic liver disease or cancer in any site. HCC cases and LC cases were separately compared to the above 139 referents in the present cohort-based case–referent study.

For cases, information on the job performed and the corresponding entry/exit dates was obtained from company files; for referents, these data were obtained through the occupational history collected by OHS occupational physicians during the medical surveillance program. Using a job–exposure matrix developed by Pirastu et al. (1991), we estimated cumulative VCM exposure by summing across the calendar years of exposure the product of the average level of VCM exposure in a job (parts per million) and years worked in that job. The variable was split into four classes using the quartiles (160, 500, and 2,500 ppm × years); it was also dichotomized (cut point, 2,500 ppm × years) when examining interactions of VCM exposure with alcohol consumption or viral hepatitis infection.

We ascertained alcohol consumption in cases and referents through hospital clinical records and/or health surveillance records. The measure was computed in grams of ethanol per day. The variable was split into three classes using 30 and 60 g/day as cut points; it was also dichotomized [cut point, 60 g/day, a threshold considered necessary for alcohol-mediated injury (Donato et al. 2002)] in examining interactions between VCM exposure and alcohol consumption.

HBV and HCV chronic infection was determined in cases and in referents by serologic markers [HBV surface antigen (HbsAg) and anti-HCV antibodies]. At analysis, the variable was coded 1 in the presence of markers for HBV and/or HCV, and 0 otherwise.

We defined “age” as the age reached by each subject in 1999 (cases and referents) or at death (cases only).

Interval variables were analyzed using Student’s *t*-test and frequency variables analyzed using Fisher’s exact test. At univariate analysis, the odds ratio (OR) and the exact 95% confidence interval (CI) were estimated using the StatXact statistical package (Mehta and Patel 1999). When a variable was broken down into classes, the lowest class was the reference subgroup at a conventional risk of 1.0. We also calculated the chi-square test for linear trend across ordered categories as described by Breslow and Day (1980).

In order to evaluate interactions of VCM exposure with alcohol intake or HBV/HCV infections, we used the OR for joint exposure (OR_AB_), the OR for exposure to a single factor (OR_A_), and the OR for exposure to the other single factor (OR_B_) to calculate the *S* synergy index as *S* = (OR_AB_ − 1) ÷ [(OR_A_ + OR_B_) − 2] (Rothman 1986). The AP_AB_ proportion of disease attributable to the interaction was calculated as AP_AB_ = (*S* − 1) ÷ *S*. A multiple logistic model was used to evaluate departure from additivity, in which terms for confounding factors were also included (Rothman 1986).

Alcohol consumption (× 10), cumulative VCM exposure (× 1,000), and HBV/HCV carrier status were used as independent variables in two models of stratified logistic regression analysis (three strata of birth year), where the dependent variable was 1 for cases (either HCC or LC) and 0 for referents (always the same 139 referents). Conditional maximum likelihood estimate ORs with exact 95% CIs and two-tailed probability of error were obtained using the LogXact statistical package (Mehta and Patel 2002).

## Results

Table 1 shows the general characteristics of 13 HCC cases, 40 LC cases, and 139 referents. With respect to referents, cases were born earlier (but they were younger because of early death), were more exposed, and drank more alcohol. The prevalence of drinkers was 92.3% (12 of 13), 97.5% (39 of 40), and 73.4% (102 of 139) in HCC, LC, and referents, respectively. The prevalence of HBV/HCV carriers was 23.1% (3 of 13), 17.5% (7 of 40), and 2.2% (3 of 139), in HCC, LC, and referents, respectively.

Table 2 shows the results at univariate analysis. The first two quartiles of cumulative exposure collapsed because of missing HCC cases. Increasing levels of cumulative VCM and alcohol consumption significantly increased the risks of HCC and LC. With VCM exposure, the trend was steeper for HCC than for LC, whereas the contrary occurred with alcohol consumption. The surprisingly high risk of HCC and LC in subjects consuming > 60 g/day of alcohol suggested an interaction with occupational exposure. Viral hepatitis infection significantly increased the risk of HCC and LC.

Table 3 shows a joint classification [by cumulative VCM exposure lower or higher than 2,500 ppm × years and *a*) alcohol consumption lower or higher than 60 g/day or *b*) viral hepatitis infection absent or present] of HCC cases and referents. The conventional risk of subjects unexposed to both of two risk factors (reference category) being 1.0, the OR estimating the effect of joint exposure to VCM and alcohol was one order of magnitude greater than the ORs estimating the effect of each factor in the absence of the other. Accordingly, the synergy index, which was close to 7, indicated a departure from an additive relation. The proportion of HCC attributable to the interaction of VCM exposure and alcohol consumption was as high as 85%. The joint effect from VCM exposure and viral hepatitis infection seemed less than multiplicative, and *S* indicated only a moderate departure from an additive relation; the interaction of two factors was responsible for 38% of the HCC cases.

Table 4 shows a joint classification [by cumulative VCM exposure lower or higher than 2,500 ppm × years and *a*) alcohol consumption lower or higher than 60 g/day or *b*) viral hepatitis infection absent or present] of LC cases and referents. The conventional risk of subjects unexposed to both risk factors being 1.0, the OR among subjects jointly exposed to VCM and alcohol was close to the product of ORs in those exposed to each factor in the absence of the other. The synergy index of 5 indicated a departure from an additive relation, and the proportion of disease among those with both exposures was 80%. The joint effect from VCM exposure and viral hepatitis infection (close to the sum of separate effects), and the *S* index (close to unity) indicated an additive relation.

Table 5 shows that by stratifying by tertiles of the birth year of the cases, holding constant the influence of HBV/HCV infection and alcohol intake, each extra increase of 1,000 ppm × years involved a 71% excess of HCC risk or a 37% excess of LC risk.

## Discussion

At the beginning of the trial, the company granted compensation for any disease to all employees, without ascertaining its occupational origin. Detailed information on this was given by the labor union and the local media during the trial (Mastrangelo et al. 2003). From 1975 (when a cross-sectional study was carried out) onward (during their employment at the VCM plant), these workers underwent yearly medical surveillance, which included liver function tests. Using such records, all subjects with liver function alteration or liver disease were identified in the course of trial. Finally, mortality and incidence registers were scrutinized. It is therefore reasonable to assume that all cases of HCC and LC occurring in the cohort were collected.

If exposure in referents had been higher than, similar to, or lower than that in the whole cohort, the HCC/LC risk would have been underestimated, valid, or overestimated, respectively. It is therefore important to consider whether our method for selecting referents may have introduced a bias. The mean ± SD of cumulative VCM was 1367.5 ± 2209.1 ppm × years in our 139 referents and 1751.5 ± 2564.8 ppm × years in the remaining 504 VCM cohort workers in the medical surveillance program. This difference is not statistically significant (*t* = 1.61; *p* = 0.11). It is therefore reasonable to deduct that ours is a cohort-based case–referent study, in which the selection of referents did not lead to an underestimation or overestimation of the risk of HCC and LC. Nor was our study affected by selection (healthy worker effect), information (diagnosis of diseases based on death certificate), or confounding biases (alcohol intake, HBV/HCV carrier status).

The main finding of the present study is that VCM exposure is an independent risk factor for the development of HCC and LC. The association between VCM exposure and HCC was suggested in early studies showing the coexistence of nodules of angiosarcoma and hepatocarcinoma in histologic liver specimens. Jones and Smith (1982) found angiosarcoma, hepatocarcinoma, and hepatoadenoma nodules in a worker who had been exposed to high doses of VCM for several years. Furthermore, Evans et al. (1983) reported the association of cirrhosis, angiosarcoma, and hepatocarcinoma in a subject exposed to VCM. This finding was confirmed by experimental studies in rats, in which VCM exposure induced both angiosarcomas and hepatocarcinomas, the two tumor types found in the same animal (Froment et al. 1994). A recent study reported on 18 HCC patients with long-term exposure to VCM; all 18 patients lacked any further identifiable risk factors for developing HCC (Weihrauch et al. 2000). Confirmatory evidence has been reported in a recent meta-analysis combining the European and North American cohorts of VCM workers (Boffetta et al. 2003): liver cancers other than angiosarcoma resulted in a meta-SMR of 1.35 (95% CI, 1.04–1.77).

A multiplicative effect between VCM exposure and alcohol in hepatocarcinogenesis was found in an experimental study (Radike et al. 1981). However, the present study is, to our knowledge, the first to report a synergistic effect between VCM exposure in humans and alcohol consumption in the development of HCC and its associated preneoplastic condition, LC. An attributable proportion of nearly 80% indicates that VCM exposure and alcohol intake have little effect separately but, in association, produce most of the disease. This may explain why the relationship between HCC (or LC) and VCM has been overlooked in epidemiologic settings (where HCC cases would be in excess only if alcohol intake were high in VCM-exposed workers) and clinical settings (where nonoccupational causes of disease are often present).

The biologic interaction between VCM and alcohol during hepatocarcinogenesis may be due to several mechanisms. Alcohol is prevalently metabolized in the liver by the microsomal ethanol-oxidizing system (MEOS) and alcohol dehydrogenase, leading to the generation of acetaldehyde and reactive oxygen species (ROSs). Chronic alcohol consumption is associated with an increased activity of MEOS, which involves the specific P450 cytochrome CYP2E1 (Lieber and DeCarli 1970). An important feature of CYP2E1 is its capacity to convert different xenobiotics into highly toxic metabolites. VCM is primarily metabolized in the liver by CYP2E1 (Stickel et al. 2002) to chloroethylene oxide and chloracetaldehyde, metabolites that can react with DNA bases and promote mutations in bacterial and mammalian cells (Marion and Boivin-Angele 1999; Marion et al. 1996; Zhou et al. 2003). Thus, ethanol induction of CYP2E1 could contribute to hepatocarcinogenesis by enhancing the conversion of VCM into toxic intermediates. The induction of CYP2E1 is also responsible for an increased catabolism of retinoic acid (Leo and Lieber 1985). The reduction of the hepatic concentration of retinoids has been shown to be associated with an up-regulation of the AP-1 (c-*jun* and c-*fos*) transcriptional complex, leading to enhanced cellular proliferation (Wang et al. 1998). By sustaining parenchymal hyperproliferation, alcohol (or viral infection) may act as a promoter in VCM carcinogenesis. Acetaldehyde is highly toxic and mutagenic and evidence has accumulated that acetaldehyde is responsible for alcohol associated carcinogenesis (Stickel et al. 2002). ROSs promote lipid peroxidation and react with DNA, resulting in alterations of DNA structure. Besides these (carcinogenetic) effects, ethanol and acetaldehyde could also enhance VCM genotoxicity through the inhibition of DNA–adduct removal (Singletary et al. 2004).

In a cohort nested case–referent study, 18 cases of liver cancer and 68 referents matched for age and specific plant of employment were selected from among 3,293 workers from six polyvinyl chloride polymerization factories in Taiwan (Wong et al. 2003). Eighty-nine percent of cancer cases had a history of HBV infection, and none of the subjects was a habitual alcohol drinker. With respect to subjects unexposed to both risk factors, the OR was 396.0 (95% CI, 22.6 to infinity) among subjects jointly exposed (high VCM exposure and viral hepatitis infection). The latter OR was greater than the product of ORs in those exposed to each factor in the absence of the other, suggesting a synergistic effect. By contrast, we found only an additive effect of VCM cumulative exposure with viral hepatitis on the risk of HCC while controlling for alcohol consumption. In our cases the prevalence of drinkers was > 90%, and the prevalence of HBV/HCV carriers was about 20%; whether the conflicting results might be explained by the different distribution of risk factors in the two working populations is unclear.

Although the mechanism whereby VCM exposure and viral hepatitis infection act additively is unknown, one hypothesis is that both factors induce liver fibrosis and regeneration, which act as a tumor promoter in hepatocarcinogenesis (Blendis et al. 2000; Pinzani 1999).

It is widely accepted that exposure to increased concentrations of VCM causes liver fibrosis (Popper and Thomas 1975). Hepatic fibrogenesis, a dynamic tissue repair process, is characterized by the increased synthesis of extracellular matrix components and changes in the perisinusoidal space (Pinzani 1995). If the noxious agent persists, liver fibrosis progresses to cirrhosis. The rate of progression of fibrosis varies greatly from patient to patient, and epidemiologic studies have identified several cofactors related to the host. Alcohol consumption is an important factor, with a detrimental effect on liver fibrosis. The activation of hepatic stellate cells is the common pathway to liver fibrogenesis, and *in vitro* studies have shown that acetaldehyde, a highly reactive toxic product of alcohol metabolism, can directly induce collagen gene transcription and promote liver fibrosis, even in the absence of necro-inflammatory changes (Moshage et al. 1990). Likewise, because of its structural similarities, the VCM metabolite chloracetaldehyde could directly sustain the progression of liver fibrosis (Larson and Bull 1991), thus explaining our finding of an increased risk of LC after exposure to high doses of VCM only. As suggested by several studies performed in human hepatic stellate cells [reviewed by Parola and Robino (2001)], reactive aldehydes are able to directly induce pro-collagen type I and III gene and protein expression with a mechanism involving nuclear translocation and activation of c-Jun amino-terminal kinase (Parola et al. 1998).

Chronic alcohol consumption decreases glutathione levels (Shaw et al. 1983), a reductive tripeptide, which inactivates both the VCM hepatotoxic metabolites chloroethylene oxide and chloracetaldehyde. Thus, VCM exposure and alcohol intake may have a hyper-additive effect in the progression to LC either because of their intrinsic hepatotoxicity and profibrogenetic activity or because they compete and/or deplete the reductive detoxification system.

HBV and HCV cause chronic liver disease, which can progress into cirrhosis. However, not all hepatitis patients have this complication, and genetic factors, alcohol (Wiley et al. 1998), and obesity (Naveau et al. 1997) may play a role. VCM exposure could contribute to the development of LC by the same mechanisms described for the alcohol–hepatitis interaction.

In conclusion, according to our findings, VCM exposure appears to be an independent risk factor for HCC and LC interacting synergistically with alcohol consumption and additively with viral hepatitis infection. This could be relevant for new prevention strategies in high-risk individuals.

## Figures and Tables

**Table 1:** VCM cumulative exposure, alcohol consumption, demographic variables, and prevalence of HBV/HCV infection in HCC cases, LC cases, and referents (Ref).

				*p*-Value*^a^*	
	HCC cases	LC cases	Ref	HCC vs. Ref	LC vs. Ref
No. of cases	13	40	139		
VCM exposure (ppm × years)	4223.8 ± 2888.4	2845.3 ± 3041.7	1367.5 ± 2209.1	< 0.001	0.001
Alcohol (g/day)	90.8 ± 62.2	108.5 ± 53.2	29.1 ± 31.6	< 0.001	< 0.001
Year of hire	1960.5 ± 3.7	1961.8 ± 6.2	1964.9 ± 6.6	0.022	0.010
Year of birth	1933.2 ± 4.0	1930.9 ± 7.7	1935.5 ± 6.5	0.196	0.002
Age at death/end of follow-up	58.8 ± 4.5	59.6 ± 7.9	63.5 ± 6.5	0.013	0.010
HBsAg/HCV positive (%)	23.1	17.5	2.2	0.009	0.001

Values shown are mean ± SD except where indicated.

a *p*-Values for a two-tailed test (Student’s *t*-test for interval variables and Fisher’s exact test for frequency variable).

**Table 2:** HCC and LC risks in relation to cumulative VCM exposure, alcohol consumption, and viral hepatitis infection at univariate analysis.

	Cases (*n*)	Ref (*n*)	OR	95% CI	χ^2^ for trend
HCC					
VCM cumulative exposure					
< 500 ppm × years	1	78	Reference		
500–2,500 ppm × years	3	37	6.32	0.48–336.0	
> 2,500 ppm × years	9	24	29.3*^#^*	3.61–1,298	16.1*^#^*
Alcohol consumption					
< 30 g/day	1	82	Reference		
30–60 g/day	4	46	7.13	0.67–355.0	
> 60 g/day	8	11	59.6*^#^*	6.51–2,676	24.3*^#^*
HBsAg/HCV					
Negative	10	136	Reference		
Positive	3	3	13.6*	1.55–111.0	
LC					
VCM cumulative exposure					
< 160 ppm × years	7	38	Reference		
160–500 ppm × years	7	40	0.95	0.26–3.51	
500–2,500 ppm × years	9	37	1.36	0.47–3.72	
> 2,500 ppm × years	17	24	3.95**	1.56–9.98	8.06**
Alcohol consumption					
< 30 g/day	1	82	Reference		
30–60 g/day	7	46	12.5*	1.50–569	
> 60 g/day	32	11	238*^#^*	31.2–9,820	78.1*^#^*
HBsAg/HCV					
Negative	33	136	Reference		
Positive	7	3	9.62**	2.03–59.6	

Ref, referents.

* *p* < 0.05,

** *p* < 0.01, and

# *p* < 0.001 by two-tailed *t*-test.

**Table 3:** Distribution of HCC cases and referents (Ref) according to a joint classification (VCM exposure and alcohol consumption or viral hepatitis infection).

	VCM < 2,500 ppm × years		VCM > 2,500 ppm × years
Alcohol < 60 g/day			
Cases/Ref	1/105		4/23
OR*^a^* (95% CI)	Reference		18.8* (1.62–218.0)
Alcohol > 60 g/day			
Cases/Ref	3/10		5/1
OR*^a^* (95% CI)	42.9** (3.41–540.0)		409*^#^* (19.6–8553.0)
Alcohol summary		*S* = 6.83; AP = 85%	
HbsAg/HCV negative			
Cases/Ref	3/113		7/23
OR*^b^* (95% CI)	Reference		25.0** (2.77–226.0)
HbsAg/HCV positive			
Cases/Ref	1/2		2/1
OR*^b^* (95% CI)	106.9** (4.43–2578.0)		210.3** (7.13–6203.0)
HbsAg/HCV summary		*S* = 1.61; AP = 38%	

Abbreviations: AP, proportion of disease attributable to interaction; *S*, Rothman’s synergy index for interaction.

a OR adjusted for age and viral hepatitis infection.

b OR adjusted for age and alcohol use.

* *p* < 0.05,

** *p* < 0.01, and

# *p* < 0.001 by two-tailed *t*-test.

**Table 4:** Distribution of LC cases and referents (Ref) according to a joint classification (VCM exposure and alcohol consumption or viral hepatitis infection).

	VCM < 2,500 ppm × years		VCM > 2,500 ppm × years
Alcohol < 60 g/day			
Cases/Ref	3/105		5/23
OR*^a^* (95% CI)	Reference		6.64* (1.03–42.8)
Alcohol > 60 g/day			
Cases/Ref	20/10		12/1
OR*^a^* (95% CI)	144.1*^#^* (24.1–860.0)		752.7*^#^* (55.3–10248.0)
Alcohol summary		*S* = 5.05; AP = 80%	
HbsAg/HCV negative			
Cases/Ref	20/113		13/23
OR*^b^* (95% CI)	Reference		8.22* (1.57–43.0)
HbsAg/HCV positive			
Cases/Ref	3/2		4/1
OR*^b^* (95% CI)	67.2** (5.14–877.0)		80.5** (3.67–1763.0)
HbsAg/HCV summary		*S* = 1.08; AP = 7%	

Abbreviations: AP, proportion of disease attributable to interaction; *S*, Rothman’s synergy index for interaction.

a OR adjusted for age and viral hepatitis infection.

b OR adjusted for age and alcohol use.

* *p* < 0.05,

** *p* < 0.01, and

# *p* < 0.001 by two-tailed *t*-test.

**Table 5:** HCC and LC risks in relation to cumulative VCM exposure, alcohol consumption, and viral hepatitis infection.

	OR (95% CI)	*p*-Value
HCC		
VCM exposure (ppm × years × 1,000)	1.71 (1.29–2.44)	0.0008
Alcohol consumption (g/day × 10)	1.36 (1.18–1.62)	< 0.0001
HBsAg/HCV positive	46.6 (1.79–4960.0)	0.0141
LC		
VCM exposure (ppm × years × 1,000)	1.37 (1.13–1.69)	0.0009
Alcohol consumption (g/day × 10)	1.70 (1.44–2.01)	< 0.0001
HBsAg/HCV positive	33.9 (3.66–410.0)	0.0007

Estimates were obtained by means of conditional regression analysis for stratified data (strata, tertiles of year of birth): OR, exact 95% CI, and exact error probability (*p*-value) for a two-tailed test.

## References

[b1-ehp0112-001188] Blendis L, Wong F, Sherman M (2000). Interferon therapy prevents hepatocellular carcinoma in some patients with chronic HCV: the role of fibrosis. Gastroenterology.

[b2-ehp0112-001188] Boffetta P, Matisane L, Mundt KA, Dell LD (2003). Meta-analysis of studies of occupational exposure to vinyl chloride in relation to cancer mortality. Scand J Work Environ Health.

[b3-ehp0112-001188] Breslow NE Day NE eds. 1980. Statistical Methods in Cancer Research. Volume 1: The Analysis of Case-control Studies. IARC Sci Publ 32. 7216345

[b4-ehp0112-001188] Donato F, Tagger A, Gelatti U, Parrinello G, Moffetta P, Albertini A (2002). Alcohol and hepatocellular carcinoma: the effect of lifetime intake and hepatitis virus infections in men and women. Am J Epidemiol.

[b5-ehp0112-001188] Du CL, Wang JD (1998). Increased morbidity odds ratio of primary liver cancer and cirrhosis of the liver among vinyl chloride monomer workers. Occup Environ Med.

[b6-ehp0112-001188] Evans DMD, Williams WJ, Kung IT (1983). Angiosarcoma and hepatocellular carcinoma in vinyl chloride workers. Histopathology.

[b7-ehp0112-001188] Froment O, Boivin S, Barbin A, Bancel B, Trepo C, Marion MJ (1994). Mutagenesis of ras proto-oncogenes in rat liver tumors induced by vinyl chloride. Cancer Res.

[b8-ehp0112-001188] IARC 1987. Overall Evaluations of Carcinogenicity: An Updating of IARC Monographs Volumes 1 to 42. IARC Monogr Eval Carcinog Risks Hum(suppl. 7). 3482203

[b9-ehp0112-001188] Jones DB, Smith PM (1982). Progression of vinyl chloride induced hepatic fibrosis to angiosarcoma of the liver. Br J Ind Med.

[b10-ehp0112-001188] Larson JL Bull RJ 1991. Hepatotoxic and hepatocarcinogenetic effects of chlorinated ethylenes. In: Hepatoxicology (Meeks RG, Harrison SD, Bull RJ, eds). Boca Raton, FL:CRC Press, 569–592.

[b11-ehp0112-001188] Lee FI, Smith PM, Bennett B, Williams DM (1996). Occupationally related angiosarcoma of the liver in the United Kingdom 1972–1994. Gut.

[b12-ehp0112-001188] Leo MA, Lieber CS (1985). New pathway for retinol metabolism in liver microsomes. J Biol Chem.

[b13-ehp0112-001188] Lieber CS, DeCarli LM (1970). Hepatic microsomal ethanol-oxidizing system: in vitro characteristics and adaptive properties in vivo. J Biol Chem.

[b14-ehp0112-001188] Marion MJ, Boivin-Angele S (1999). Vinyl chloride-specific mutations in humans and animals. IARC Sci Publ.

[b15-ehp0112-001188] Marion MJ, De Vivo I, Smith S, Luo JC, Brandt-Rauf PW (1996). The molecular epidemiology of occupational carcinogenesis in vinyl chloride exposed workers. Int Arch Occup Environ Health.

[b16-ehp0112-001188] Mastrangelo G, Fedeli U, Fadda E, Milan G, Turato A, Pavanello S (2003). Lung cancer risk in workers exposed to poly (vinyl chloride) dust: a nested case-referent study. Occup Environ Med.

[b17-ehp0112-001188] Mehta C Patel N eds. 1999. StatXact for Windows. Cambridge, MA:Cytel Software Corporation.

[b18-ehp0112-001188] Mehta C Patel N eds. 2002. LogXact for Windows. Cambridge, MA:Cytel Software Corporation.

[b19-ehp0112-001188] Moshage H, Casini A, Lieber CS (1990). Acetaldehyde selectively stimulates collagen production in cultured rat liver fat-storing cells but not in hepatocytes. Hepatology.

[b20-ehp0112-001188] Mundt KA, Dell LD, Austin RP, Luippold RS, Noess R, Bieglow C (2000). Historical cohort study of 10 109 men in the North American vinyl chloride industry, 1942–72: update of cancer mortality to 31 December 1995. Occup Environ Med.

[b21-ehp0112-001188] Naveau S, Giraud V, Borotto E, Aubert A, Capron F, Caput JC (1997). Excessive weight risk factor for alcoholic liver disease are likely to play a leading role in the progression to LC. Hepatology.

[b22-ehp0112-001188] Parola M, Robino G (2001). Oxidative stress-related molecules and liver fibrosis. J Hepatol.

[b23-ehp0112-001188] Parola M, Robino G, Marra F, Pinzani M, Bellomo G, Leonarduzzi G (1998). HNE interacts directly with JNK isoforms in human hepatic stellate cells. J Clin Invest.

[b24-ehp0112-001188] Pinzani M (1995). Novel insights in the biology and physiology of the Ito cell. Pharmacol Ther.

[b25-ehp0112-001188] Pinzani M (1999). Liver fibrosis. Springer Semin Immunopathol.

[b26-ehp0112-001188] Pirastu R, Baccini M, Biggeri A, Comba P (2003). Cohort study of vinyl chloride exposed workers in Porto Marghera: update of the mortality follow up. Epidemiol Prev.

[b27-ehp0112-001188] Pirastu R, Belli S, Bruno C, Comba P, De Santis M, Foa V (1991). La mortalità dei lavoratori del cloruro di vinile in Italia. Med Lav.

[b28-ehp0112-001188] Popper H, Thomas LB (1975). Alterations of liver and spleen among workers exposed to vinyl chloride. Ann NY Acad Sci.

[b29-ehp0112-001188] Radike MJ, Stemmer KL, Bingham E (1981). Effect of ethanol on vinyl chloride carcinogenesis. Environ Health Perspect.

[b30-ehp0112-001188] Rothman KJ ed. 1986. Modern Epidemiology. Boston:Little, Brown & Company.

[b31-ehp0112-001188] Ryder SD (2003). Guidelines for the diagnosis and treatment of hepatocellular carcinoma (HCC) in adults. Gut.

[b32-ehp0112-001188] Shaw S, Rubin KP, Lieber CS (1983). Depressed hepatic glutathione and increased diene conjugates in alcoholic liver disease: evidence of lipid peroxidation. Dig Dis Sci.

[b33-ehp0112-001188] Singletary KW, Barnes SL, van Breemen RB (2004). Ethanol inhibits benzo[a]pyrene-DNA adduct removal and increases 8-oxo-deoxyguanosine formation in human mammary epithelial cells. Cancer Lett.

[b34-ehp0112-001188] Stickel F, Schuppan D, Hahn EG, Seitz HK (2002). Cocarcinogenic effects of alcohol in hepatocarcinogenesis. Gut.

[b35-ehp0112-001188] Wang XD, Liu C, Chung JY, Stickel F, Seitz HK, Russel RM (1998). Chronic alcohol intake reduces retinoic acid concentration and enhances AP-1 (c-Jun and c-Fos) expression in rat liver. Hepatology.

[b36-ehp0112-001188] Ward E, Boffetta P, Andersen A, Colin D, Comba P, Deddens JA (2001). Update of the follow-up of mortality and cancer incidence among European workers employed in the vinyl chloride industry. Epidemiology.

[b37-ehp0112-001188] Weihrauch M, Lehnert G, Kockerling F, Wittekind C, Tannapfel A (2000). p53 Mutation pattern in hepatocellular carcinoma in workers exposed to vinyl choride. Cancer.

[b38-ehp0112-001188] Wiley TE, McCarthy M, Breidi L, McCarthy M, Layden TJ (1998). Impact of alcohol on the histological and clinical progression of hepatitis C infection. Hepatology.

[b39-ehp0112-001188] Wong RH, Chen TC, Wang JD, Du CL, Cheng TJ (2003). Interaction of vinyl chloride monomer exposure and hepatitis B viral infection on liver cancer. J Occup Environ Med.

[b40-ehp0112-001188] Zhou H, Josephy PD, Kim D, Guengerich FP (2003). Functional characterization of four allelic variants of human cytochrome P450 1A2. Arch Biochem Biophys.

